# Puberty Blocker, Leuprolide, Reduces Sex Differences in Rough-and-Tumble Play and Anxiety-like Behavior in Juvenile Rats

**DOI:** 10.1210/endocr/bqae046

**Published:** 2024-04-10

**Authors:** Gabriela de Faria Oliveira, Amber T Nguyen, Leykza Carreras-Simons, Thomas Niepsuj, Salma H Gadelhak, Aimee K Johnson, Ashwakh Abdalla, Eden Lev, Sofia G Torres Román, Samantha N Fuchs, Joan S Jorgensen, Walid A Farhat, Anthony P Auger

**Affiliations:** Wisconsin National Primate Research Center, Madison, WI 53715, USA; Department of Psychology, University of Wisconsin–Madison, Madison, WI 53706, USA; Department of Psychology, University of Wisconsin–Madison, Madison, WI 53706, USA; Department of Psychology, University of Wisconsin–Madison, Madison, WI 53706, USA; Department of Psychology, University of Wisconsin–Madison, Madison, WI 53706, USA; Department of Psychology, University of Wisconsin–Madison, Madison, WI 53706, USA; Department of Psychology, University of Wisconsin–Madison, Madison, WI 53706, USA; Department of Psychology, University of Wisconsin–Madison, Madison, WI 53706, USA; Department of Psychology, University of Wisconsin–Madison, Madison, WI 53706, USA; Department of Psychology, University of Wisconsin–Madison, Madison, WI 53706, USA; Department of Psychology, University of Wisconsin–Madison, Madison, WI 53706, USA; Department of Comparative Biosciences, School of Veterinary Medicine, University of Wisconsin–Madison, Madison, WI 53706, USA; Division of Pediatric Urology, School of Medicine and Public Health, University of Wisconsin–Madison, Madison, WI 53705, USA; Department of Psychology, University of Wisconsin–Madison, Madison, WI 53706, USA

**Keywords:** leuprolide, GnRH, puberty blockers, adolescent, behavior

## Abstract

We examined the effect of the puberty blocker, leuprolide acetate, on sex differences in juvenile rough-and-tumble play behavior and anxiety-like behavior in adolescent male and female rats. We also evaluated leuprolide treatment on gonadal and pituitary hormone levels and activity-regulated cytoskeleton-protein messenger RNA levels within the adolescent amygdala, a region important both for rough-and-tumble play and anxiety-like behavior. Our findings suggest that leuprolide treatment lowered anxiety-like behavior during adolescent development, suggesting that the maturation of gonadotropin-releasing hormone systems may be linked to increased anxiety. These data provide a potential new model to understand the emergence of increased anxiety triggered around puberty. Leuprolide also reduced masculinized levels of rough-and-tumble play behavior, lowered follicle-stimulating hormone, and produced a consistent pattern of reducing or halting sex differences of hormone levels, including testosterone, growth hormone, thyrotropin, and corticosterone levels. Therefore, leuprolide treatment not only pauses sexual development of peripheral tissues, but also reduces sex differences in hormones, brain, and behavior, allowing for better harmonization of these systems following gender-affirming hormone treatment. These data contribute to the intended use of puberty blockers in stopping sex differences from developing further with the potential benefit of lowering anxiety-like behavior.

The use of gonadotropin-releasing hormone (GnRH) agonists (GnRHas), colloquially known as puberty blockers, is to suppress puberty and treat gender dysphoria: a sense of misalignment with one's biological sex and gender identity that may occur in transgender and nonbinary (TNB) adolescents. The use of many GnRHas has been proven to be safe and approved by the US Food and Drug Administration in the early 1990s (1) for the treatment of precocious puberty in adolescents (2), and reproductive cancers in adults (3). Therefore, it is important to understand the effect of pausing puberty on the brain and behavioral development during adolescence.

Puberty is triggered, in part, from increased pulsatile release of GnRH. The rhythmic secretion of GnRH from the hypothalamus act on GnRH receptors to trigger the release of follicle-stimulating hormone (FSH) and luteinizing hormone (LH) from the pituitary (4), which, in turn, causes the release of factors such as estrogens from the ovaries and androgens from the testes. Variations in the ratio of androgens and estrogens result in sexual differentiation of the brain, physiology, and behavior (5, 6). GnRHas modulate this pathway, leading to desensitization of GnRH receptors. As a result, the release of FSH and LH from the anterior pituitary gland is suppressed, gonadal release of estrogens and androgens is lowered, and the development of secondary sexual characteristics is delayed (6). While pubertal development is paused using puberty blockers, studies indicate that pubertal development proceeds in a typical manner on withdrawal of puberty blockers. For example, recent studies in rats indicate that while reproductive physiology and behavior are delayed with the usage of leuprolide acetate, these are later restored following cessation of the puberty blockade (7, 8).

In addition to the physiological changes associated with puberty, this developmental period is also associated with dramatic shifts in mental health risk, such as increased vulnerability to anxiety, depression, and suicidal ideation (9-11), which are exacerbated TNB adolescents (12). These symptoms are likely attributed to gender dysphoria, social exclusion, inadequate parental support, and discrimination experienced by TNB youth (10). Recent studies have shown that those receiving gender-affirming health care exhibit lower levels of anxiety, depression, and suicidal ideation (13, 14); however, it is not fully clear if this is due to increased social support, halting the biological underpinnings of increased mental health risk following puberty, or both.

The present study examines the physical and psychological effects of daily leuprolide treatment on adolescent development in male and female rats. We investigated the effects of a commonly used GnRHa, leuprolide acetate, on rough-and-tumble play and anxiety-like behavior. The effect of the GnRHa on hormone levels and activity-regulated cytoskeletal-protein (ARC) expression were also evaluated. The ARC protein is an inducible transcription factor associated with increased synaptic plasticity and has been reported to be expressed at higher levels in females compared to males (15). Therefore, we used ARC as a marker to determine if GnRHas altered general activity related to synaptic plasticity (16) and sex differences within the developing amygdala.

## Materials and Methods

### Animals

Juvenile Sprague Dawley Rats (female = 24, male = 24) were purchased from Charles River Laboratories and kept at the University of Wisconsin–Madison Department of Psychology animal facility. All experimental procedures were approved by the University of Wisconsin–Madison Animal Care and Use Committee in accordance with US National Institutes of Health guidelines. Animals were group-housed, 4 per cage, with same-sex and same-treatment cage mates in polycarbonate cages under standard laboratory conditions: reverse light/dark cycle of 12 hours/12 hours, food and water ad libitum. Wood shavings were used for bedding and plastic bones were placed in each cage for enrichment.

### Treatment of Animals

Starting on postnatal day (PD) 27 (juvenile), 24 male and 24 female rats (categorized by external genitalia) were assigned to receive either leuprolide acetate (25 µg/kg dissolved in 0.9% sterile physiological saline; female: n = 12; male n = 12) or sterile physiological saline (1 mL/kg 0.9% NaCl; female: n = 12; male: n = 12). Subjects received subcutaneous injections daily starting at 8 Am between PD 27 to PD 39, that is, a juvenile, peripubertal, individual (17). Body weight was measured daily and vaginal opening for female subjects were measured to evaluate the effectiveness of leuprolide on pubertal delay.

### Rough-and-Tumble Play Behavior

Juvenile social play behavior was assessed from PD 27 to PD 37 using paradigms adapted from those previously reported (18, 19). Recordings of home-cage behavior were made twice for 5 minutes at hours 2 and 4 after the lights were off. For each recording day, every animal was marked with a permanent marker on the back and tail within 3 hours before lights off. Recordings were made by replacing the cage lid with Plexiglass and moving the cage to a table under a night vision camera. Cages were left beneath the camera for 5-minute trials for a total observation time of 110 minutes per cage. Rough-and-tumble play behavior was assessed by scoring the frequency and duration of rats engaging in pouncing, pinning, chasing, and boxing (19). Pouncing is where one animal jumps or lunges at another animal. Pinning is where one animal is on top of another, and the other animal is on its back (20). Boxing is scored as 2 animals on hind legs engaging each other with their 4 paws. Counts and duration across all observation windows were summed and analyzed by 2 observers unaware of group assignments.

### Anxiety-like Behavior

Anxiety-like behavior was evaluated using the elevated plus maze test on days PD 38 and 39. The elevated plus maze is a black acrylic structure standing 50 cm off the floor and consisting of 4 arms (2 opposing open and 2 opposing closed arms). Open arms are 50 cm length × 10 cm wide. Closed arms are 50 cm length × 10 cm width with 39-cm high walls (21). The rats were placed in the center of the maze, where the arms intersect, facing an open arm, and the exploratory behavior within the maze was recorded for 5 minutes. An experimenter unaware of the treatment groups scored the recordings. Parameters quantified were the number of entries into the open and closed arms and the total time spent in the open and closed arms and the center chamber. An entry was counted when all 4 paws crossed into a particular portion of the maze.

### Tissue Collection

On PD 40, rats were anesthetized using isoflurane. Brains were immediately removed, and flash-frozen in isopentane on dry ice, and then stored at −80 °C until sectioned. Trunk blood was collected in serum separator tubes (BD 365967) and centrifuged at 4500 rpm for 8 minutes at 4 °C. Serum was aliquoted and stored at −80 °C until assayed. Tissue samples were homogenized, and total RNA and DNA were collected using an AllPrep DNA/RNA Mini Kit (Qiagen; catalog No. 80204) according to manufacturer directions. RNA purity and concentrations were measured using a NanoDrop 2000 spectrophotometer (ThermoScientific; catalog No. ND-2000LAPTOP). RNA was converted to complementary DNA (cDNA) using the High-Capacity RNA-to-cDNA Kit (Thermo Fisher; catalog No. 4387406) using manufacturer directions. Real-time quantitative polymerase chain reaction (qPCR) was conducted using the Stratagene Mx3000PTM real-time PCR system, and cDNA was amplified with PowerUp SYBR Green Master Mix (Thermofisher; catalog No. A25741). After amplification, dissociation curves were created to ensure purity of the PCR products and run on a gel to confirm a single band of approximate weight. Primers were targeted to activity-regulated cytoskeleton-associated protein (Arc; NM_019361): forward primer TGAAGCAGCAGACCTGAC and reverse primer GAGTCATGGAGCCGAAGTC, ribosomal protein L13A (Rpl13a; NM_173340): forward primer AGCAGCTCTTGAGGCTAAGG and reverse primer GGGTTCACACCAAGAGTCCA and TATA-box binding protein (Tbp; NM_00 L 004198): forward primer TGTGAAGTTCCCCATAAGGC and reverse primer GGAGAACAATTCTGGGTTTGATC. Primers for ARC and TBP were purchased as predesigned primers from IDT DNA technologies; whereas those for Rpl13a were designed as published previously (22). Relative fold cDNA levels were calculated using the 2 −ΔΔCt method normalized to the average of 2 reference genes (*Rpl13a* and *Tbp*).

### Hormones

Serum samples were sent to Wisconsin National Primate Research Center assay services for steroid analysis. Testosterone, progesterone, corticosterone, estradiol, estrone, and 17-OH progesterone levels were assayed by liquid chromatography–tandem mass spectrometry using a Sciex 6500+ Mass Spectrometer. Levels were detected in ng/mL concentrations. Levels of adrenocorticotropin (ACTH), brain-derived neurotrophic factor (BDNF), follicle-stimulating hormone (FSH), growth hormone (GH), luteinizing hormone (LH), prolactin, and thyrotropin (TSH) were also measured. A total of 50 μL of serum per sample was assayed in duplicates using a MILLIPLEX MAP Rat Pituitary Magnetic Bead Panel—Endocrine Multiplex Assay (catalog No. RPTMAG-86 K) with a BioPlex 200 system. Levels were detected in ng/mL concentrations.

### Statistics

All data were collected by researchers unaware of group classification. Behavioral, hormonal, and qPCR data were analyzed with 2-way ANOVAs and the Tukey post hoc tests. We also performed Pearson correlations to evaluate the association between ARC messenger RNA (mRNA) levels, testosterone, and frequency of pinning behavior. All statistical analyses were performed using both SigmaPlot 13.0 and GraphPad Prism. We used the Grubbs test for outliers with GraphPad Prism version 10.0.2 for MacOS (GraphPad Software). All values are written as mean ± SEM. Significance is set at a *P* value of less than .05.

## Results

### Puberty and Body Weight

Leuprolide treatment significantly delayed vaginal opening in female subjects (*P* = .0002; *t* = 4.829). As expected, body weight increased with age (female: *P* < .0001; F [11 264] = 138.7; male: *P* < .0001; F [11 264] = 231.6); however, there were no weight differences between leuprolide and control treatment.

### Anxiety-Like Behavior

**Figure 1.**
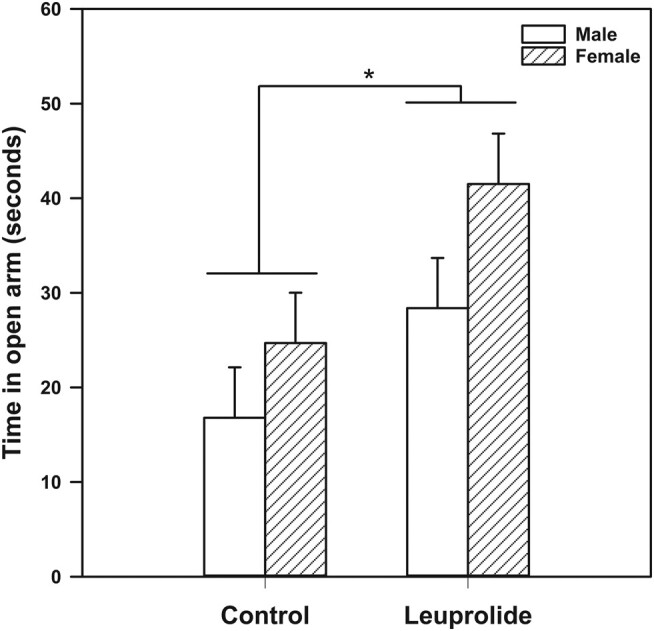
Effects of leuprolide anxiety-like behavior using the elevated plus maze. Total time in seconds within the open arm. Leuprolide decreased anxiety-like behavior in male and female rats (as shown by higher duration in the open arm). Subjects received subcutaneous injections from postnatal day (PD) 27 to PD 39. Anxiety-like behavior was evaluated on days PD 38 and 39. Statistical comparisons were performed using 2-way analysis of variance followed by Tukey post hoc tests. Data are presented as mean values ± SEM. *P less than or equal to .05.

Leuprolide treatment significantly reduced anxiety-like behavior in male and female rats. Specifically, leuprolide-treated animals spent more time in the open arm in comparison to the control group (F (1, 44) = 7.103; *P* = .011; Fig. 1).

### Rough-and-Tumble Play Behavior

**Figure 2.**
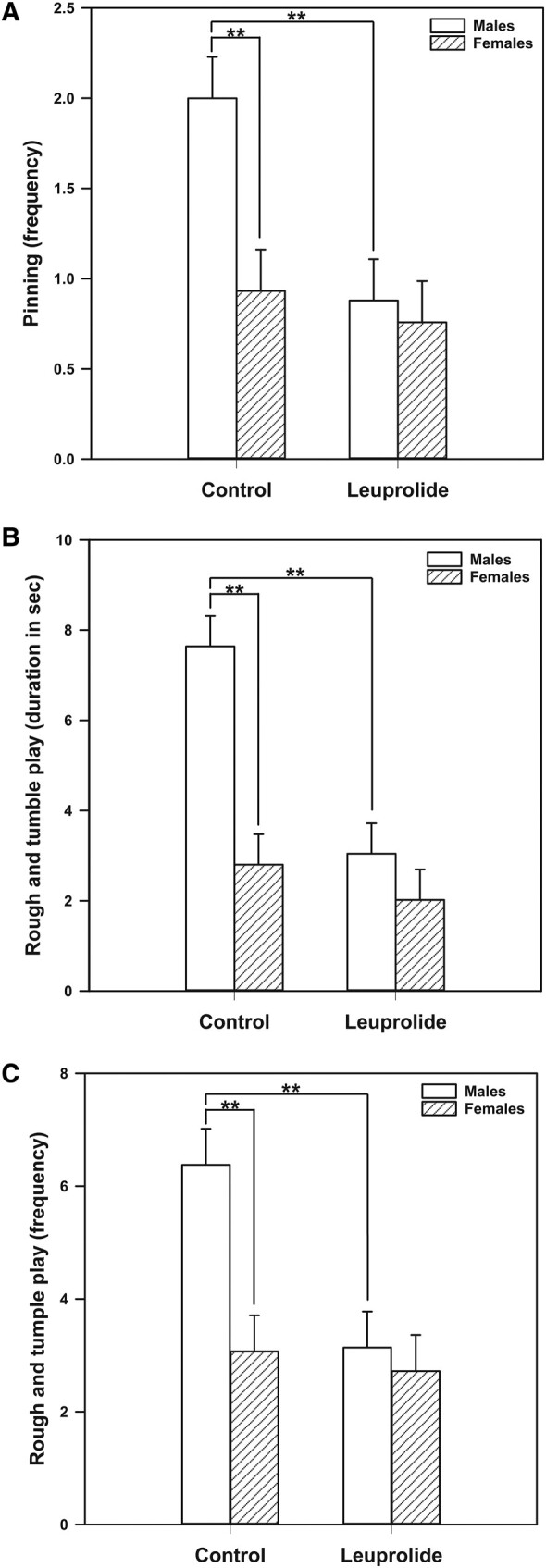
Effects of leuprolide treatment on rough-and-tumble play behavior. A, Pinning frequency; B, total rough-and-tumble play behavior duration; and C, frequency were lowered in males to resemble females. Subjects received subcutaneous injections from postnatal day (PD) 27 to PD 39. Play behavior was evaluated from PD 27 to 37. Statistical comparisons were performed using 2-way analysis of variance followed by Tukey post hoc tests. Data are presented as mean values ± SEM. **P less than or equal to .01.

A 2-way ANOVA indicated the main effects of biological sex (F [1, 44] = 8.469; *P* = .006) and treatment (F [1, 44] = 7.861; *P* = .007), as well as an interaction of biological sex and treatment (F [1, 44] = 5.106; *P* = .029) on rough-and-tumble play behavior frequency (Fig. 2C). As expected, post hoc analysis with a Tukey test indicated that male control rats engaged in higher levels of total rough-and-tumble play behavior frequency compared with control female rats (*P* < .001). Daily treatment with leuprolide acetate eliminated the sex difference in rough-and-tumble play behavior frequency. That is, leuprolide-treated males exhibited lower levels of rough-and-tumble behavior compared to control males (*P* < .001), resembling that of control females. Interestingly, leuprolide treatment did not alter rough-and-tumble play behavior frequency in females.

We also found significant differences in the duration of rough-and-tumble play (see Fig. 2B) with main effects of both biological sex (F [1, 44] = 18.947; *P* < .001) and leuprolide treatment (F [1, 44] = 15.940; *P* < .001), as well as an interaction between biological sex and treatment (F [1, 44] = 6.967; *P* = .011). Tukey post hoc comparisons indicate that control males engage in a longer duration of rough-and-tumble play compared with females (*P* < .001). Furthermore, leuprolide-treated males exhibit lower levels of play duration compared with control males (*P* < .001) and engaged in play levels that were similar to control females. Leuprolide treatment did not alter the duration of rough-and-tumble play in females.

We also examined effect of leuprolide treatment on pinning behavior (see Fig. 2A), as this is the most consistent component of rough-and-tumble play that is higher in males in comparison to females. Specifically, we found main effects of biological sex (F [1, 44] = 6.754; *P* = .013) and leuprolide treatment on pinning (F [1, 44] = 8.012; *P* = .007), as well as interactions between biological sex and leuprolide treatment for pinning (F [1, 44] = 4.281; *P* = .044). Tukey post hoc analysis indicates that control males engaged in higher levels of pinning compared to females (*P* = .002), and that leuprolide treatment lowered these behaviors to female-typical levels (*P* = .001).

### Hormones

We measured the effect of leuprolide on FSH, LH, testosterone, estradiol, progesterone, corticosterone, as well as ACTH, BDNF, growth hormone (GH), prolactin, and TSH.

**Figure 3.**
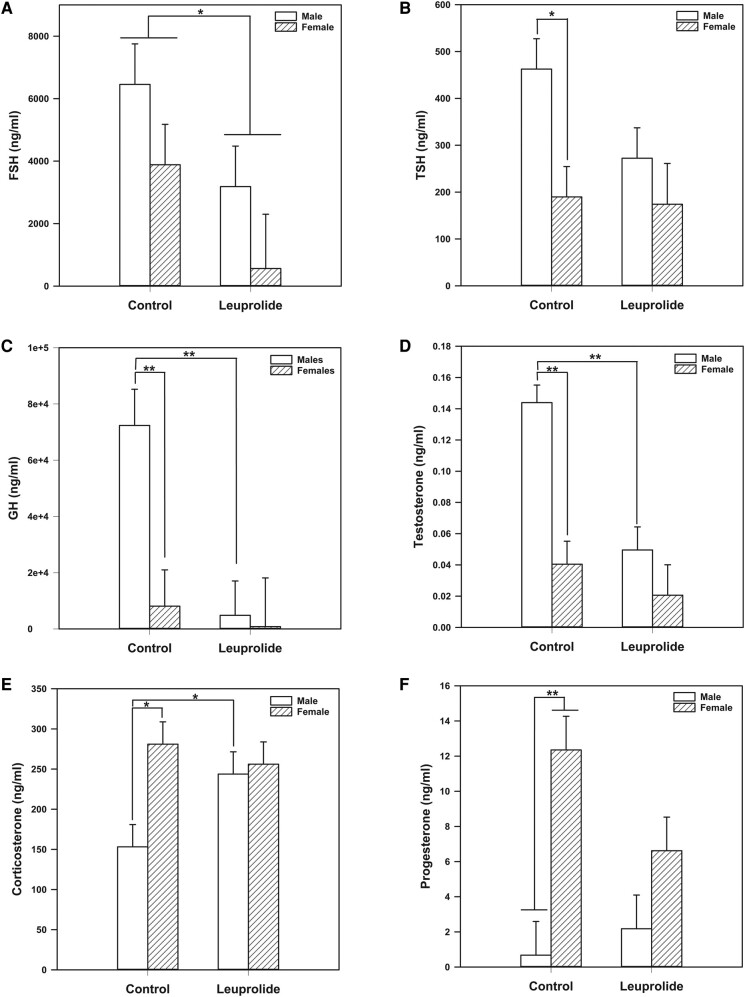
Changes in hormone levels among control and leuprolide rats. A, Leuprolide lowered follicle-stimulating hormone (FSH) levels both in male and female rats. Leuprolide lowered B, thyrotropin (TSH), C, growth hormone (GH), and D, testosterone in males to female levels. E, Corticosterone increased in leuprolide-treated males to female levels. F, Leuprolide trended lower levels of progesterone in males to female levels. Subjects received subcutaneous injections from postnatal day (PD 27) to PD 39. Serum samples were collected on PD 40. Statistical comparisons were performed using 2-way analysis of variance followed by Tukey post hoc tests. Data are presented as mean values ± SEM. *P less than or equal to .05; **P less than or equal to .01.

Using a 2-way ANOVA, we found a main treatment effect was leuprolide lowering FSH levels (F [1, 28] = 5.390; *P* = .028) Fig 3A.

For TSH levels, there was a main effect of biological sex (F [1, 28] = 6.824; *P* = .014). Tukey post hoc comparisons indicate that control males had higher levels of TSH compared to control females (*P* = .006). Additionally, the Tukey post hoc comparisons found that leuprolide males had lower TSH levels than control males (*P* = .048) (see Fig. 3B).

For GH, there was a main effect of biological sex (F [1, 29] = 5.965; *P* = .021) and leuprolide treatment (F [1, 29] = 7.164; *P* = .012). Furthermore, there was also an interaction between biological sex and leuprolide treatment (F [1, 29] = 4.651; *P* = .039). The Tukey post hoc comparisons showed that within the control group, males had higher GH levels than females (*P* = .002). Leuprolide lowered GH in males (*P* < .001) to levels that were similar to control females. Leuprolide had no effect on females, suggesting it only eliminated the sex difference in GH levels (Fig 3C).

For testosterone, there was a main effect of biological sex (F [1, 26] = 18.642; *P* < .001) and leuprolide treatment (F [1, 26] = 13.856; *P* = < .001). There was also an interaction between biological sex and leuprolide treatment (F [1, 26] = 5.898; *P* = .022). The Tukey post hoc comparisons showed that control males had higher levels of testosterone compared to control females (*P* < .001). Leuprolide treatment significantly reduced testosterone levels in males (*P* < .001) to levels that resembled control females (Fig 3D).

For corticosterone, we found a main effect of biological sex (F [1, 44] = 6.390; *P* = .015). There was an additional interaction between biological sex and treatment (F [1, 44] = 4.339; *P* = .043). The Tukey post hoc comparisons indicate that there was a sex difference in corticosterone levels (*P* = .002), with control females having higher levels than control males. Leuprolide treatment increased corticosterone levels in males (*P* = .026) to female typical levels (Fig 3E).

For progesterone, there was a main effect of biological sex (F [1, 44] = 17.668; *P* < .001), with females having higher levels compared with males. While the interaction between biological sex and leuprolide treatment was not statistically significant (*P* = .066), there was a trend in which leuprolide-treated females experienced lower levels of progesterone compared to control females (Fig 3F).

There was no effect of treatment or biological sex on estradiol, estrone, prolactin, BDNF, or ACTH levels (*P* > .05). For LH, several samples (27%) were below detectability of the assay, preventing analyses from being conducted.

### Amygdala Messenger RNA Levels

**Figure 4.**
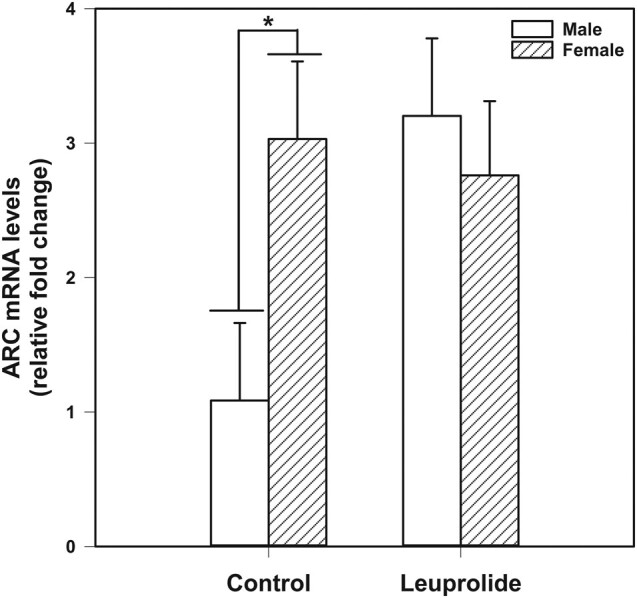
Amygdala activity-regulated cytoskeletal-protein (ARC) messenger RNA (mRNA) levels. Control females have higher levels of ARC mRNA compared to control males. Additionally, leuprolide-treated males have higher mRNA levels of ARC than control males. Statistical comparisons were performed using 2-way analysis of variance followed by Tukey post hoc tests. Data are presented as mean values ± SEM.* *P* less than or equal to .05.

For ARC mRNA levels (Fig. 4), there was a significant interaction between biological sex and leuprolide treatment (F [1, 41] = 9.617; *P* = .003). Tukey post hoc comparisons indicated that control females had higher levels of ARC mRNA compared to control males (*P* = .006). There was also a treatment effect within males, with leuprolide males having higher mRNA levels of ARC than control males (*P* = .001), eliminating the sex difference in ARC mRNA levels.

### Association Between Activity-regulated Cytoskeletal-Protein Messenger RNA, Testosterone, and Pinning Behavior

**Figure 5.**
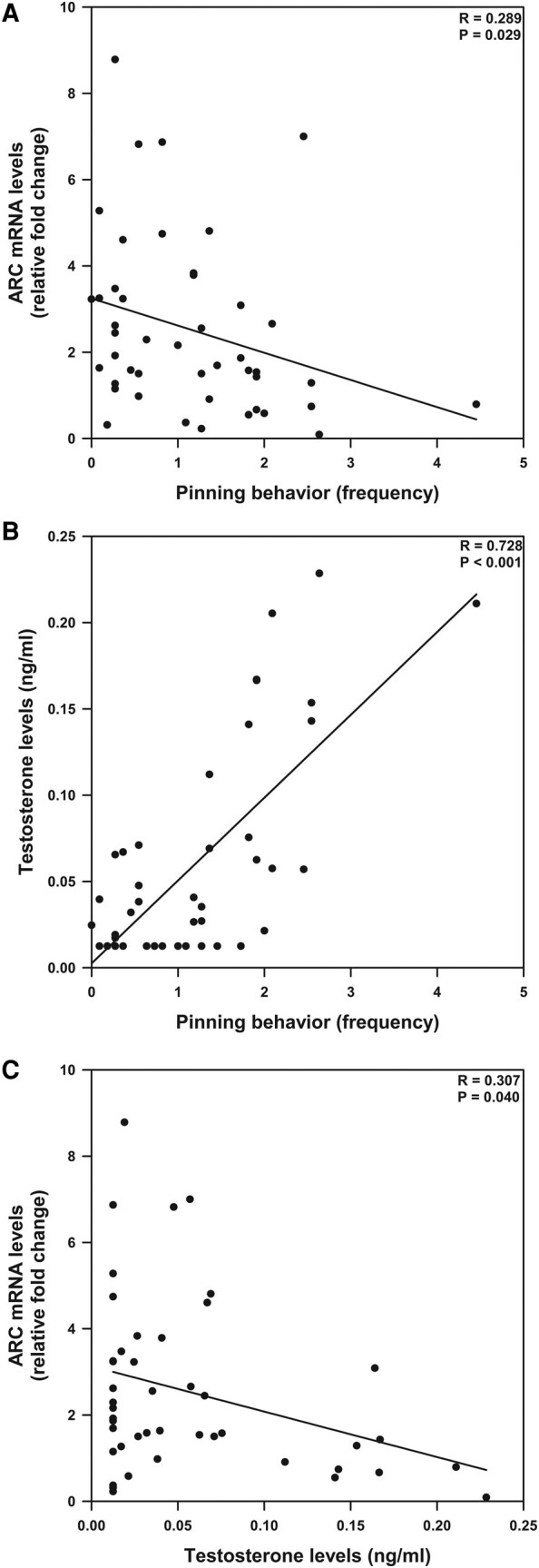
Association between activity-regulated cytoskeletal-protein (ARC) messenger RNA (mRNA), testosterone, and pinning behavior. A, ARC mRNA levels were negatively correlated with frequency of pinning behavior. B, Testosterone levels were positively associated with pinning behavior. C, ARC mRNA levels were also negatively correlated with testosterone levels.

ARC mRNA levels were negatively correlated with frequency of pinning behavior (*P* = .029; *R* = 0.289; Fig. 5A). Testosterone levels were positively associated with pinning behavior (*P* < .001; *R* = 0.728; Fig. 5B). ARC mRNA levels were negatively correlated with testosterone levels (*P* = .040; *R* = 0.307) Fig. 5C).

## Discussion

Our findings indicate that treatment with the puberty blocker, leuprolide acetate, reduces sex differences in behavior, hormones, and physiology in adolescent rats. More important, leuprolide treatment reduced anxiety-like behavior both in adolescent male and female rats. These findings suggest that while leuprolide treatment had the expected findings of delaying sexual differentiation in adolescent physiology, it also reduced the development of sex differences in rough-and-tumble behavior, as well as alleviating anxiety-like symptoms. Specifically, leuprolide-treated males mirrored the female rough-and-tumble behavioral phenotype and both males and females treated with leuprolide had lower anxiety-like behavior compared to the controls.

### Anxiety-like Behavior

It is known that human adolescent development is associated with increased anxiety and depression (23). These symptoms can be exacerbated in transgender individuals experiencing gender dysphoria when secondary sexual characteristics develop during puberty that do not align with their gender identity. As such, TNB youth may seek gender-affirming health care to reduce symptoms of gender dysphoria. Though research on mental health in transgender youth is somewhat sparse compared to those who do not identify as transgender or nonbinary, studies examining the effects of gender-affirming health care found that TNB adolescents receiving gender-affirming hormone therapy had reduced anxiety and depression symptoms (11). It is unclear if the reduction in anxiety levels is a physiological consequence of gender-affirming hormone therapy, or a result of the psychosocial support and psychological relief of puberty suppression. Our findings in a rat model allow us to eliminate the social confounders and directly assess the effect of leuprolide treatment on anxiety. Specifically, our data suggest that leuprolide may directly lower anxiety during adolescent development both in male and female rats. Currently, it is not known if leuprolide is directly lowering anxiety-like behavior or if it is disrupting the increased anxiety-like behavior that occurs following puberty. Our model provides a potential pathway to investigate the onset of adolescent anxiety, but longitudinal studies must be conducted to determine if the reductions in anxiety-like behavior due to puberty blockade are permanent.

### Rough-and-Tumble Play Behavior

Leuprolide treatment also eliminated sex differences in rough-and-tumble play behavior during adolescent development. Consistent with previous studies, we found that control male rats engaged in higher levels of rough-and-tumble play behavior compared to female rats (18). This sex difference in rough-and-tumble play behavior was observed both in frequency and duration of play. Leuprolide treatment reduced rough-and-tumble play behavior in males to female control levels. Leuprolide treatment had no effect on females, indicating that leuprolide treatment only eliminated the sex difference in juvenile rough-and-tumble play behavior. Additionally, as males engage in more rough-and-tumble play behavior, leuprolide treatment appeared to block its masculinization.

One potential mechanism by which leuprolide alters rough-and-tumble play behavior is through the modulation of steroid hormone production. It has been established that testosterone plays a role in the organization of rough-and-tumble play behavior. Testosterone levels in males are higher than females at birth and during adolescence (24), which is replicated in the observed levels of testosterone in our control rodents. Previous research has shown that high levels of androgen exposure around birth increases the level of rough-and-tumble play behavior during the juvenile period (25). However, researchers also found that castration during the juvenile period did not seem to reduce play behavior in males (19). Likewise, when juvenile female rats were exposed to androgens, rough-and-tumble play behavior was not increased. Therefore, it is believed that androgen exposure during the early neonatal time period is responsible for later juvenile rough-and-tumble play behavior, and that juvenile hormone levels are not involved in its regulation (18, 25). In the present study, we found that leuprolide reduced juvenile rough-and-tumble play behavior in males and lowered testosterone levels. Leuprolide treatment did not alter these measures in female rats. Therefore, treatment with leuprolide eliminated the sex difference in rough-and-tumble play behavior. We also found that the reduction in testosterone levels correlates with reduced rough-and-tumble play behavior. This suggests that gonadal hormones may play some role in rough-and-tumble play behavior or that GnRH systems play a permissive role during this time period. That is, neonatal testosterone still plays an important role in organizing the neurocircuitry of play behavior but disruption of adolescent GnRH signaling pathways appears to halt the sex difference in play behavior at this time.

### Glycoprotein and Steroidal Hormones

As predicted, leuprolide treatment lowered FSH levels both in males and females. Consistent with previous findings (26), we found that leuprolide treatment reduced testosterone levels. We also found that progesterone levels were higher in females than males. While there was a trend toward leuprolide-treated females experiencing lower levels of progesterone than control females, it did not reach statistical significance. Additionally, we found an overall sex difference in TSH, where control males had higher levels of TSH compared to control females. It is notable that leuprolide lowered TSH in males to female-typical levels. Leuprolide had no effect on TSH levels in females. Therefore, these data suggest that leuprolide treatment eliminates the sex differences in TSH levels and does not necessarily push males to typical hypothyroidism levels but reduces it to female-typical levels. Indeed, previous research suggests that GnRHa reduced TSH levels within 2 weeks in humans but did not alter 3,5,3′-triiodothyronine or thyroxine levels (27). Corticosterone levels followed an inverse pattern of males having lower levels of corticosterone than females. Specifically, leuprolide increased male corticosterone levels to female levels, effectively eliminating the sex difference in corticosterone levels. The inverse relationship between corticosterone and testosterone levels in rodents has been exemplified in previous research studies. Moreover, increased corticosterone levels have been correlated to the inhibition of testosterone biosynthesis (28). Furthermore, castration results in increased corticosterone levels. Therefore, it is plausible that the administration of leuprolide acetate, which reduces testosterone secretion, elevates corticosterone levels (29). Finally, we found that GH levels were higher in males than females and leuprolide treatment eliminated this sex difference, reducing male levels to female-typical levels. While GH is released in pulses and varies depending on time of day, previous findings have reported that peak levels at PD 30 and 90 are higher in males compared with females (30). As testosterone positivity regulates GH release (31), it is likely that reduced testosterone levels may account for lower GH levels following leuprolide treatment. Together, these findings suggest that leuprolide effectively eliminates many of the sex differences in hormone levels during this developmental period.

Leuprolide had no effect on levels of estradiol or estrone in males or females. Previous studies have shown that the profile of estrogen levels between males and females are similar throughout the juvenile and prepubescent periods (24). As circulating estradiol levels are low, they may not actively be secreted by the ovaries at sufficient levels to be modulated by leuprolide. Together with the aforementioned data, leuprolide treatment appears to eliminate observed sex differences in hormone levels but has few effects on hormones that are not different between the sexes at this stage of development.

### Activity-regulated Cytoskeletal-Protein Messenger RNA Levels in the Amygdala

We found a sex difference in ARC mRNA levels within the amygdala, whereby control females exhibited higher ARC mRNA levels than control males. Leuprolide increased ARC mRNA levels in males to female levels and thereby eliminated the sex difference in ARC mRNA levels. Our findings are consistent with previous studies indicating that female rats had significantly higher ARC mRNA levels than male rats (15). We found a negative correlation between ARC levels and testosterone levels: Those presenting lower ARC levels had higher testosterone levels. As testosterone levels are a driving moderator in the duration of rough-and-tumble play behavior, we investigated the relationship between ARC, testosterone, and pinning behavior (a reliable scoring category for sex difference in rough-and-tumble play behavior). Our findings show that rats that had lower levels of ARC had increased pinning behavior frequencies. Last, we conducted a linear regression between testosterone and pinning behavior and found that rats with higher levels of testosterone exhibited increased frequency of pinning behavior. Therefore, lower testosterone levels correlate with increased ARC mRNA levels within the amygdala, which is associated with reduced pinning behavior. Previous research has shown that females have higher levels of neurogenesis within the newborn amygdala (30), and recent data suggest that males have greater attrition of newborn cells within the amygdala around puberty (32). These data indicate that sex differences in markers of synaptic plasticity are found across development; however, it remains to be investigated why ARC is associated with lower levels of play. ARC plays an important role in plasticity and learning, and rough-and-tumble play behavior has been associated with the development and maturation of neural circuits in the amygdala (33). Rather than eliminating play behavior and its social benefits in these rodents, leuprolide eliminated sex differences, allowing male rats to express a more female-typical phenotype in this developmental period. Together, our data indicate that leuprolide exposure eliminates sex differences across several domains, underscoring its intended use in gender-affirming health care.

### Conclusion

In this study, we found leuprolide treatment had a significant effect on male and female adolescent rats in reducing anxiety-like behavior, suggesting a potential direct link between GnRH system maturation and the emergence of anxiety during adolescent development. Leuprolide treatment also eliminated sex differences in rough-and-tumble play behavior, effectively preventing the masculinization of this behavior in males. The correlation between reduced testosterone levels and diminished rough-and-tumble play behavior highlights the role of gonadal hormones influencing these behaviors. These findings are crucial for understanding the potential benefits of leuprolide in gender-affirming health care, as it may aid in reducing behaviors that contribute to gender dysphoria and social distress. Leuprolide removed sex differences in hormone levels, affecting testosterone, TSH, and corticosterone. Leuprolide exposure led to increased ARC mRNA levels in male rats, eliminating the sex difference observed in control rats. This suggests that leuprolide pauses further masculinization of some systems within the amygdala and may contribute to a more feminized behavioral phenotype during this developmental period. Additional studies are needed to examine if these effects are reversable or somewhat permanent. It is also pertinent to observe that several of our observations appear to affect males rather than females. At the developmental stage in our paradigm there is a notable disparity in the levels of testosterone and estradiol (24). Testosterone is significantly higher in males than females on PD 40; whereas estradiol has just started to be released (34). If we had continued the treatment longer, it is likely we would have observed more dramatic effects in females when estradiol levels are further increasing.

These findings hold clinical relevance, particularly for TNB youth receiving gender-affirming health care, whose anxiety and depression symptoms can be heightened throughout pubertal and adolescent development. They highlight the potential benefits of leuprolide treatment in gender-affirming healthcare. They underscore the capacity of leuprolide to reduce anxiety and eliminate sex differences in behavior and hormone levels, aligning with its intended role in delaying puberty and pausing sexual development. These results offer support for the use of puberty blockers to prevent further development of sex differences and alleviate anxiety-like symptoms in adolescents, particularly in a clinical setting. To fully understand the clinical effect, further research, including longitudinal studies, is necessary to assess the long-term effects of leuprolide treatment on these outcomes.

## Data Availability

Original data generated and analyzed during this study are included in this published article or in the data repositories listed in “References.”

## Abbreviations

ACTH: adrenocorticotropin
ANOVA: analysis of variance
ARC: activity-regulated cytoskeletal-protein
BDNF: brain-derived neurotrophic factor
cDNA: complementary DNA
FSH: follicle-stimulating hormone
GH: growth hormone
GnRH: gonadotropin-releasing hormone
GnRHas: gonadotropin-releasing hormone agonists
LH: luteinizing hormone
mRNA: messenger RNA
PD: postnatal day
qPCR: quantitative polymerase chain reaction
TBP: TATA-box binding protein
TNB: transgender and nonbinary
TSH: thyrotropin
